# Inhibition of DAI refrains dendritic cells from maturation and prolongs murine islet and skin allograft survival

**DOI:** 10.3389/fimmu.2023.1182851

**Published:** 2023-05-01

**Authors:** Pengrui Cheng, Qian Jian, Zongli Fu, Ronghai Deng, Yi Ma

**Affiliations:** ^1^ Organ Transplant Center, The First Affiliated Hospital, Sun Yat-sen University, Guangzhou, China; ^2^ Guangdong Provincial Key Laboratory of Organ Donation and Transplant Immunology, The First Affiliated Hospital, Sun Yat-sen University, Guangzhou, China; ^3^ Guangdong Provincial International Cooperation Base of Science and Technology (Organ Transplantation), The First Affiliated Hospital, Sun Yat-sen University, Guangzhou, China

**Keywords:** dendritic cells, DNA-dependent activator of IFN regulatory factors, immunosuppression, islet transplant, skin transplant, adenovirus vector

## Abstract

**Introduction:**

Central to allograft rejection is the T cell-mediated adaptive immune response initiated by activated dendritic cells (DCs). Previous studies have shown that the DNA-dependent activator of IFN regulatory factors (DAI) is involved in the maturation and activation of DCs. Therefore, we hypothesized that inhibition of DAI could prevent DCs from maturation and prolong murine allograft survival.

**Methods:**

Donor mouse bone marrow-derived dendritic cells (BMDCs) were transduced with the recombinant adenovirus vector (AdV-DAI-RNAi-GFP) to inhibit DAI expression (DC-DAI-RNAi), and the immune cell phenotype and function of DC-DAI-RNAi upon lipopolysaccharide (LPS) stimulation were evaluated. Then DC-DAI-RNAi was injected into recipient mice before islet transplantation and skin transplantation. The survival times of islet and skin allograft were recorded and the proportions of T cell subsets in spleen and secretion levels of cytokines in serum were measured.

**Results:**

We identified that DC-DAI-RNAi inhibited the expression of main co-stimulatory molecules and MHC-II, exhibited strong phagocytic ability, and secreted high levels of immunosuppressive cytokines and low levels of immunostimulating cytokines. Recipient mice treated with DC-DAI-RNAi had longer islet and skin allograft survival times. In the murine islet transplantation model, we observed an increase in Treg cells proportion, a reduction in Th1 and Th17 cells proportions in spleen, and similar trends in their secreted cytokines in serum in the DC-DAI-RNAi group.

**Conclusion:**

Inhibition of DAI by adenovirus transduction inhibits the maturation and activation of DCs, affects the differentiation of T cell subsets as well as their secreted cytokines, and prolongs allograft survival.

## 1 Introduction

Islet and skin transplantation have become favorable therapeutic approaches for treating Type 1 diabetes and skin wounds and burns caused by accidents, respectively (1, 2). However, acute and chronic rejection remain impediments to long-term allograft survival, requiring lifelong immunosuppressive therapy (3), which in turn increases the risk of multiple adverse reactions such as opportunistic infections and the development of neoplasms (4). Therefore, it is urgently warranted to develop new and more effective strategies to induce immune tolerance and improve allograft survival.

Central to allograft rejection is T cell-mediated adaptive immune response initiated by activated dendritic cells (DCs). DCs are the most potent antigen-presenting cells (APCs), and mature DCs (mDCs) present antigens to CD4^+^ and CD8^+^ T cells in class I or II MHC, thus inducing potent T cell responses. On the contrary, immature dendritic cells (imDCs) usually show a high endocytosis capacity and low T cell activation potential, displaying the characteristics of tolerogenic DCs (tolDCs) (5–7). Therefore, maintaining the immature state of DCs is essential for improving allograft survival.

DNA-dependent activator of IFN-regulatory factors (DAI), the first DNA sensor discovered in the cytoplasm, was initially identified in tumor stromal tissue and named DLM-1 (8). Subsequently, it was grouped among a conserved family of Z-DNA binding proteins after characterization of two N-terminal Z-DNA binding domains, and hence was termed Z-DNA binding protein 1 (ZBP1) (9). In 2007, Taniguchi and colleagues demonstrated the role of ZBP1 as a cytosolic DNA sensor that could initiate DNA-mediated innate immune response, and thus proposed the alternative name DAI (10). Recent studies have shown that DAI was up-regulated in DCs during multiple virus infections, which underscores the integral role of DAI in the maturation and activation of DCs (11, 12). However, to our knowledge, no studies have investigated the role of DAI in alloimmune response after transplantation, which may offer new insights into transplant tolerance.

In this study, we hypothesized that inhibition of DAI would prevent DCs from maturation and thus exerted immune protective effects on murine allograft. To test our hypothesis, we transduced mouse bone marrow-derived dendritic cells (BMDCs) with the recombinant adenovirus vector (AdV-DAI-RNAi-GFP) to inhibit DAI expression and verified whether DC-DAI-RNAi prolonged islet and skin allograft survival.

## 2 Materials and methods

### 2.1 Reagents

Recombinant murine granulocyte-macrophage colony-stimulating factor (GM-CSF) and interleukin (IL)-4 were purchased from PeproTech (NJ, USA). The adenoviral short hairpin RNA vector AdV-DAI-RNAi-GFP was constructed by Beijing Xbhcbio Co., Ltd. (Beijing, China). Microbead conjugated anti-CD11c and LS separation columns were purchased from Miltenyi Biotec (Bergisch Gladbach, Germany). Nylon Wool Fiber Syringe was purchased from Polysciences, Inc. (Eppelheim, Germany). Rabbit anti-mouse DAI primary antibody was purchased from Signalway Antibody LLC. (MD, USA). Mouse anti-mouse GAPDH primary antibody and goat anti-rabbit and anti-mouse secondary antibodies were purchased from Proteintech Group, Inc. (Wuhan, China). Cell Stimulation Cocktail plus Protein Transport Inhibitor, Fixable Viability Stain 780, Transcription Factor Buffer Set, rat anti-mouse fluorescence-conjugated CD45, CD3, CD4, CD8, CD25, Foxp3, interferon (IFN)-γ, IL-17, CD11c, CD80, CD86, MHC-II (I-A/I-E) monoclonal antibodies, as well as their corresponding isotype controls, were purchased from BD PharmingenTM (San Diego, CA, USA). Rabbit anti-mouse insulin and glucagon monoclonal antibodies were purchased from Abcam (Cambridge, MA, USA). Enzyme-linked immunosorbent assay (ELISA) kits for the determination of the concentrations of IL-10, IL-12, IL-17, IFN-γ and transforming growth factor (TGF)-β were purchased from Meimian Industrial Co., Ltd. (Jiangsu, China). Streptozotocin (STZ), lipopolysaccharide (LPS), Histopaque 1077, and tetramethylrhodamine-dextran (TRITC-dextran) were purchased from Sigma-Aldrich (St. Louis, CA, USA). Diphenylthiocarbazone (DTZ), acridine orange (AO), and propidium iodide (PI) were purchased from Beijing Solarbio Science & Technology Co., Ltd. (Beijing, China). Liberase TL, a blood glucose meter, and blood glucose test strips were purchased from Roche (Basel, Switzerland).

### 2.2 Animals

Male C57BL/6 mice (H-2^b^) and BALB/c mice (H-2^d^) weighing 25–30 g were purchased from the Experiment Animal Center of Sun Yat-sen University (Guangzhou, China) and maintained under specific pathogen-free (SPF) conditions. Animal experiments were approved by the Institutional Animal Care and Use Committee (IACUC) of Sun Yat-sen University (approval No (2020). 287). BALB/c mice and C57BL/6 mice were used as donors and recipients, respectively.

### 2.3 Cell culture and adenovirus vector transduction

Bone marrow precursor cells obtained from the femurs and tibias of healthy BALB/c mice were cultured in a complete culture medium (RPMI-1640 supplemented with 10% FBS, 1% penicillin-streptomycin, 20 ng/mL recombinant murine GM-CSF, and 10 ng/mL IL-4) at a concentration of 1×10^6^ cells/mL in 6-well plates. Non-adherent cells were discarded, and a new medium supplemented with recombinant murine GM-CSF (20 ng/mL) and IL-4 (10 ng/mL) was added 48 h later. On day 6 of the culture, loosely adherent cell clusters were resuspended and harvested. These bone marrow-derived immature DCs (imDCs) were then purified by immunomagnetic sorting with anti-CD11c-conjugated microbeads and transduced with the recombinant adenovirus vector AdV-DAI-RNAi-GFP (DC-DAI-RNAi) or AdV-GFP (DC-GFP) at a multiplicity of infection (MOI) of 1:100 for 48 h in serum-free RPMI 1640. mDCs were generated by stimulating imDCs with LPS (1 µg/mL) for 48 h. T cells were isolated from healthy C57BL/6 mice spleens using the nylon wool fiber syringe.

### 2.4 Pancreatic islet transplantation

The transplant procedure was performed as previously described (13). C57BL/6 mice were injected intraperitoneally with streptozotocin (200 mg/kg) after at least 6 h of fasting to induce Type 2 diabetes mellitus (T2DM). Five days later, the blood glucose levels were regularly monitored, and mice with non-fasting blood glucose levels> 16.7 mmol/L (300 mg/dL) for three consecutive days were regarded as diabetic recipient mice. The islets were isolated from BALB/c mice. Briefly, the pancreas was harvested and digested in the thermostatic water bath at 37°C after reverse perfusion of Liberase TL through the common bile duct and then purified by density gradient centrifugation. Before transplantation, DTZ staining and AO-PI dual staining were performed to evaluate the purity and viability of isolated islets, respectively. The C57BL/6 recipient mice were randomly divided into three groups: the DC-DAI-RNAi group, the DC-GFP group, and the control group, receiving DC-DAI-RNAi, DC-GFP and phosphate buffered saline (PBS), respectively. One day before islet transplantation, approximately 2×10^6^ DCs were injected into diabetic recipient mice through the tail vein. On transplantation day, approximately 300 islets were transplanted into the subcapsular space of the recipient mice’s kidney using a glass capillary tube probe. Islet transplantation was considered curative when the postoperative non-fasting blood glucose level was <11.1 mmol/L (200 mg/dL), while the rejection of an islet allograft was defined as non-fasting blood glucose level >16.7 mmol/L (300 mg/dL) for at least two consecutive days.

### 2.5 Intraperitoneal glucose tolerance test

To test glucose tolerance of the recipient mice, intraperitoneal injections of glucose solution (2 g/kg) were performed after 4-6 h of fasting on postoperative day (POD) 10 (we observed that about half of the islet allografts in the DC-GFP group rejected at this time). Blood glucose levels were determined every 15 mins for 120 mins using a glucose meter through samples collected from the tail tips. The islet-bearing kidney was then removed for hematoxylin-eosin (HE) and insulin/glucagon immunohistochemistry (IHC) staining on POD 11, and then IPGTT was repeated to validate the therapeutic efficacy of islet allografts.

### 2.6 Skin transplantation

The transplant procedure was performed as previously described (14). Briefly, full-thickness skin allografts with an area of 1 cm×1 cm were isolated from the tails of BALB/c mice. C57BL/6 recipient mice were randomly divided into three groups: the DC-DAI-RNAi group, the DC-GFP group and the control group, and they received DC-DAI-RNAi, DC-GFP and equivalent PBS *via* caudal vein injections on the day before transplantation, respectively. At surgery, skin allografts were transplanted onto the backs of the recipient mice. Vaseline gauzes and pressure bandages were used to cover and fix the skin allografts for 4 days. From day 3 onward, allografts were evaluated daily for skin necrosis in a blinded fashion. Allograft rejection was defined as necrosis of more than 80% of skin tissue.

### 2.7 Western blot analysis

Total protein was extracted from DCs using a lysis buffer (Epizyme Biotech, China) containing 10% phosphatase inhibitor and 1% protease inhibitor. Protein concentration was determined using a bicinchoninic acid (BCA) kit (Thermo Scientific, USA) according to the manufacturer’s instructions. A total of 20 μg total protein from DC-DAI-RNAi and DC-GFP were separated on a 12% sodium dodecyl sulfate-polyacrylamide gel electrophoresis (SDS-PAGE) and transferred onto a nitrocellulose membrane pretreated with methanol. The membranes were incubated overnight at 4°C with anti-mouse DAI and GAPDH primary antibodies and subsequently incubated with the corresponding secondary antibodies for 1 h. The bands were imaged using ECL color development, and band gray values were analyzed by ImageJ software.

### 2.8 Flow cytometry analysis

Purified imDCs, DC-DAI-RNAi and DC-GFP were stimulated with LPS for 48 h, stained with fluorochrome-conjugated anti-mouse CD80, CD86 and MHC-II antibodies, and then analyzed by flow cytometry (BD LSRFortessa X-20, USA). In the murine islet transplantation model, splenic single cell suspension of recipient mice in each group were harvested on POD 11 and on the day of allograft rejection, they were stained with fluorochrome-conjugated anti-mouse CD45, CD3, CD4, CD25, IL-17, IFN-γ, and Foxp3 antibodies and then analyzed by flow cytometry. For intracellular cytokine staining, the cells were cultured with Cell Stimulation Cocktail plus Protein Transport Inhibitor for 4-6 h, fixed and permeabilized with Transcription Factor Buffer Set according to the manufacturer’s protocols. Th1 cells were defined as CD3^+^/CD4^+^/IFN-γ^+^, Th17 cells as CD3^+^/CD4^+^/IL-17^+^, and Treg cells as CD4^+^/CD25^+^/Foxp3^+^. Data were analyzed using FlowJo software.

### 2.9 ELISA for cytokine detection

Cytokines in the culture supernatants of DC-DAI-RNAi and DC-GFP upon LPS stimulation for 48 h were measured using mouse IL-10, IL-12, IFN-γ, and TGF-β ELISA kits according to the manufacturer’s instructions. In the murine islet transplantation model, blood samples were collected from the retro-orbital sinus of recipient mice on POD 11 and on the day of allograft rejection. Levels of cytokines such as IL-10, IL-17, and IFN-γ in the serum were measured by ELISA kits. The quantification of cytokine levels was based on the optical density (OD) values and the standard curve drawn through the standard samples.

### 2.10 Mixed leukocyte reaction

DC-DAI-RNAi and DC-GFP were stimulated with LPS for 48 h, incubated with mitomycin C (Abmole, US) for 2 h at 37°C, and washed three times with PBS. The pretreated DCs were co-cultured with T cells from C57BL/6 mice spleen in 96-well culture plates at ratios of 1:1, 1:10, 1:20, 1:50, and 1:100 for 72 h. Untreated T cells and RPMI-1640 with 10% FBS culture medium served as negative and blank controls, respectively. Cell Counting Kit (CCK-8, Abmole, US) was added to the medium for 4 h before the end of culture, and T cell proliferation was analyzed by determining the OD_450_ using a microplate reader (Bio-Rad, iMark™). The stimulation index (SI) was calculated according to the following formula: stimulation index = (OD_sample_ − OD_blank control_)/(OD_negative control_ − OD_blank control_). Meanwhile, MLR culture supernatants were collected from each group for concentration measurements of IL-10, IL-17, and IFN-γ using ELISA kits according to the manufacturer’s instructions.

### 2.11 Phagocytic ability analysis

The phagocytic ability of DCs was evaluated by uptaking TRITC-dextran. Purified imDCs, DC-DAI-RNAi and DC-GFP were stimulated with LPS for 48 h and incubated with TRITC-dextran (25 μg/mL) at 37°C or 4°C for 2 h. The pretreated DCs were harvested and washed three times with 4°C PBS containing 1% FBS. TRITC-positive staining cells were detected by flow cytometry.

### 2.12 Histology and IHC analysis

The islet allografts were harvested on POD 11 and on the day of allograft rejection. The skin allografts were harvested on POD 7 (about half of the skin allografts in the DC-GFP group rejected at this time) and on the day of allograft rejection. Then the allografts were processed by formalin fixation and paraffin embedment, cut into approximately 3-5 μm thick sections, and stained with HE for morphological evaluation. In addition, an IHC staining procedure was carried out on islet allografts with anti-mouse insulin and glucagon monoclonal antibodies to assess the quantity and activity of residual islets. The sample slices were evaluated in a blinded fashion.

### 2.13 Statistical analysis

All data were expressed as mean ± SD. Differences between the two groups were analyzed using the Student’s t-test. One-way ANOVA was used to compare multiple groups. The Kaplan-Meier analysis was used to assess differences between allograft survival curves and calculate the P-values. A P-value <0.05 was considered statistically significant. All statistical analyses were performed with GraphPad Prism 9.0 and SPSS 22.0 software.

## 3 Results

### 3.1 Genetically modified adenovirus vector increases the transduction efficiency of DCs

**Figure 1:**
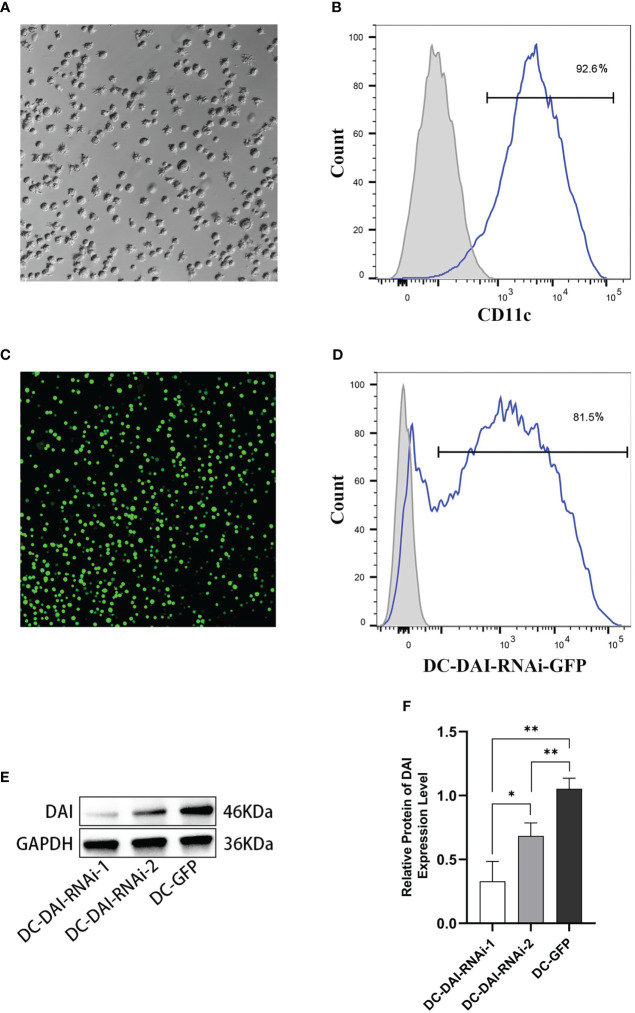
Generation of mouse bone marrow-derived dendritic cells (BMDCs) and transduction of the adenovirus vector. **(A)** Mature DCs (×200). **(B)** Purity of BMDCs (n=3). **(C)** The expression of green fluorescent protein (GFP) in DCs 48 h after AdV-DAI-RNAi-GFP transduction observed in fluorescence microscopy (×200). **(D)** Transduction efficiency of AdV-DAI-RNAi-GFP (n=3). **(E, F)** Western blot analysis of DAI protein expression levels in DCs transduced with AdV-DAI-RNAi-1 and AdV-DAI-RNAi-2 (*P < 0.05, **P < 0.01, n=3).

BMDCs were successfully generated and purified to > 90% by immunomagnetic selection (**Figures 1A, B**). Purified DCs were transduced with AdV-DAI-RNAi-GFP (DC-DAI-RNAi) or AdV-GFP (DC-GFP) at an MOI of 1:100 for 48 h. GFP fluorescence was detected by a fluorescent microscope (Olympus Fluoview FV3000, Japan) (**Figure 1C**). Analysis of GFP expression by flow cytometry showed that approximately 80% of DCs were successfully transduced with the adenovirus vector (**Figure 1D**). Moreover, the expression levels of DAI were measured by western blot to determine the inhibition efficiencies of AdV-DAI-RNAi-1 and AdV-DAI-RNAi-2. The results indicated that DAI expression levels were significantly decreased in DC-DAI-RNAi compared to those in DC-GFP, and AdV-DAI-RNAi-1 showed better inhibition efficiency than AdV-DAI-RNAi-2 (**Figure 1E, F**). We chose DC-DAI-RNAi-1 for the subsequent experiments.

### 3.2 DC-DAI-RNAi inhibits the expression of major co-stimulatory molecules and MHC-II and remains a negative immunomodulatory function

**Figure 2:**
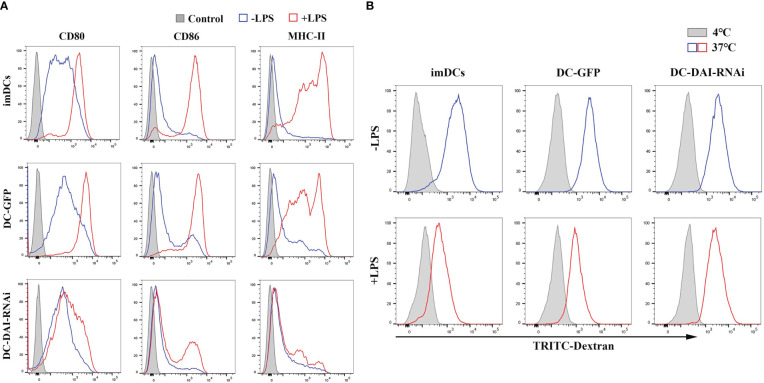
DC-DAI-RNAi inhibited the expression of major co-stimulatory molecules (CD80 and CD86) and MHC-II. **(A)** Purified imDCs, DC-GFP and DC-DAI-RNAi were stained with fluorescence-conjugated CD80, CD86 and MHC-II monoclonal antibodies. The expression of major co-stimulatory molecules (CD80 and CD86) and MHC-II in DC-DAI-RNAi were significantly inhibited compared to those of imDCs and DC-GFP upon LPS stimulation (n=3). **(B)** DC-DAI-RNAi exhibited stronger phagocytic ability after LPS stimulation (n=3).

LPS can induce DCs maturation *via* the activation of pattern-recognition receptors (PRRs) (15). To investigate the influence of inhibition of DAI expression on DCs maturation induced by LPS, purified imDCs, DC-GFP and DC-DAI-RNAi were stimulated with LPS (1 µg/mL) for 48 h, and the expression levels of major co-stimulatory molecules CD80 and CD86, as well as MHC-II, were examined using flow cytometry analysis. The results showed that DC-DAI-RNAi inhibited the expression of CD80, CD86, and MHC-II, while imDCs and DC-GFP expressed high levels of CD80, CD86, and MHC-II following LPS stimulation (**Figure 2A**). A dextran uptake assay was performed to evaluate the phagocytic ability of DCs, and the results showed that LPS stimulation significantly weakened the phagocytic capacity of both imDCs and DC-GFP, but had little impact on that of DC-DAI-RNAi (**Figure 2B**). Additionally, there was no significant difference between imDCs and DC-GFP in phenotype expression level and phagocytic ability after LPS stimulation, indicating that both imDCs and DC-GFP became mDCs upon LPS stimulation for 48 h.

**Figure 3:**
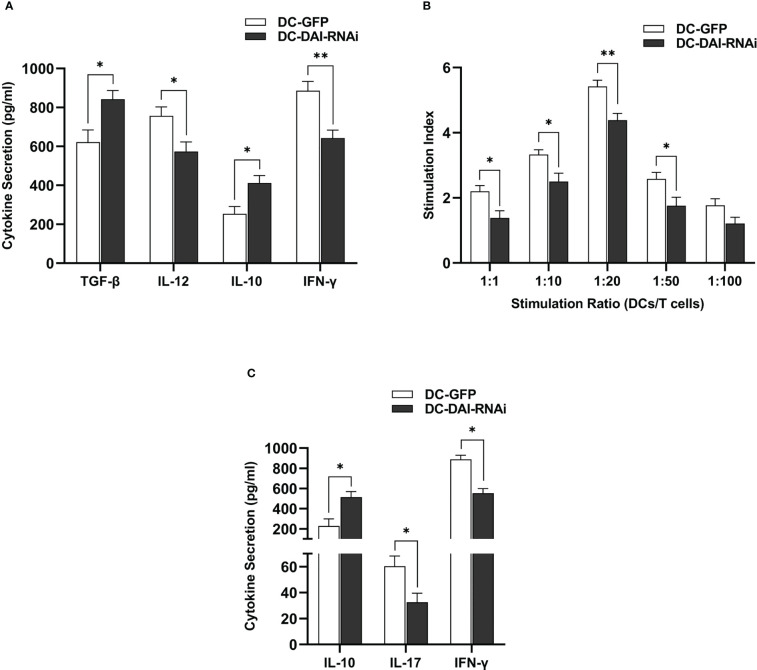
DC-DAI-RNAi exhibited the immunosuppressive properties of tolerogenic DCs (tolDCs). **(A)** DC-DAI-RNAi secreted higher levels of immunosuppressive cytokines and lower levels of immunostimulating cytokines than DC-GFP (*P < 0.05, **P < 0.01, n=5). **(B)** The capacities of DCs to stimulate T cell proliferation in different ratios were evaluated by stimulation index (SI), which was calculated according to the following formula: stimulation index = (OD_sample_ − OD_blank control_)/(OD_negative control_ − OD_blank control_) (*P < 0.05, **P < 0.01, n=5). **(C)** DC-DAI-RNAi increased IL-10 level and reduced IL-17 and IFN-γ levels in the supernatant of the mixed lymphocyte reaction (MLR) (*P < 0.05, n=5).

DCs could secrete a variety of cytokines to modulate the immune response. Hence, we compared the the levels of immunostimulating and immunosuppressive cytokines secreted by DC-DAI-RNAi and DC-GFP upon LPS stimulation, and the results indicated that DC-DAI-RNAi secreted higher levels of immunosuppressive cytokines such as IL-10 and TGF-β and lower levels of the immunostimulating cytokine IL-12 than DC-GFP (**Figures 3A**). To evaluate the capacity of DC-DAI-RNAi to stimulate T cell proliferation, LPS-pretreated DC-DAI-RNAi and DC-GFP were co-cultured with T cells at different ratios for 72 h. The results showed that T cell proliferation in the DC-GFP group was faster than in the DC-DAI-RNAi group within a certain range of DCs/T cell ratio (from 1:1 to 1:50), especially when the ratio was 1:20 (**Figures 3B**). ELISA analysis of cytokine levels in the supernatant of MLR (DCs: T cell = 1:20) showed that IL-17 and IFN-γ levels were decreased while IL-10 level was increased in the DC-DAI-RNAi group (**Figure 3C**).

Together, the results suggested that inhibition of DAI expression arrested the maturation and activation of DCs, even in the presence of maturation and activation stimuli, giving DC-DAI-RNAi the immunosuppressive properties of tolDCs.

### 3.3 DC-DAI-RNAi prolongs islet and skin allograft survival

We utilized the mouse models of islet transplantation and skin transplantation to determine the effect of DC-DAI-RNAi on transplantation immunity.

**Figure 4:**
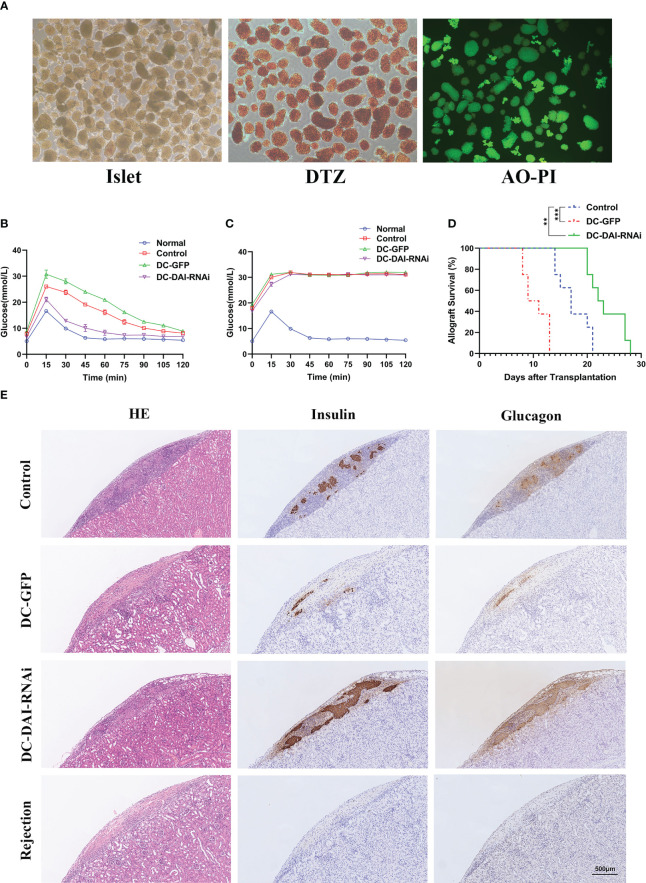
DCs transduced with AdV-DAI-RNAi-GFP prolonged islet allograft survival. **(A)** Islets isolated from perfusion, digestion, and purification procedures with sufficient purity (DTZ, ×40) and activity (AO-PI, ×40). **(B)** Intraperitoneal glucose tolerance tests (IPGTTs) were performed on postoperative day (POD) 10 to test recipient glucose tolerance after 4–6 h of fasting (n=5). **(C)** IPGTTs were repeated after the removal of the islet-bearing kidney to confirm that the recipient blood glucose response was dependent on the islet allograft (The upper limit of the blood glucose meter is 33.3 mmol/L, and if the blood glucose level was above the limit, it was recorded as 33.3 mmol/L. Data were expressed as means only, n=5). **(D)** Survival curves of islet allografts in the PBS control, DC-GFP, and DC-DAI-RNAi groups. Allograft survival among groups was compared with Kaplan–Meier analysis (**P < 0.01, ***P < 0.001, n=8). **(E)** On POD 11, islet-bearing kidneys in each group were removed and stained with the indicated markers. DC-DAI-RNAi-treated recipients displayed less lymphocyte infiltration and higher insulin and glucagon expression than the other groups, indicating greater function and better survival of islet allografts (× 200, n=5).

Before the transplantation of islets, the purity and activity of isolated islets were evaluated by DTZ staining and AO-PI staining, respectively, and the results showed that both the purity and the activity of the islets were high (**Figure 4A**). To investigate the functional activity of transplanted islet allografts *in vivo*, IPGTT (2 g/kg) was performed on POD 10 after 4-6 h of fasting to assess glucose tolerance, and glucose levels in the tail vein blood were measured every 15 min for 120 min. There was no significant difference in the baseline blood glucose levels among groups before IPGTT. After glucose injection, the blood glucose level in each group increased sharply, reached a peak at 15 mins, and then decreased with time. Furthermore, we observed a better and faster response to glucose in the DC-DAI-RNAi group than in the other groups (**Figure 4B**). The islet-bearing kidneys were then removed on POD 11, and the IPGTT was repeated. The results showed that the blood glucose response of the recipient mice was islet allograft-dependent (**Figure 4C**). The islet-bearing kidneys harvested on POD 11 and on the day of allograft rejection were stained with HE as well as anti-mouse insulin and glucagon monoclonal antibodies to assess the quantity and activity of residual islets. The results showed that islet allografts in the DC-DAI-RNAi group had better survival and activity and less lymphocyte infiltration than other groups (**Figure 4E**). The survival time of islet allografts was determined by periodic monitoring of non-fasting blood glucose levels. DC-DAI-RNAi significantly prolonged the survival time of islet allografts compared to the other groups (**Figure 4D**).

**Figure 5:**
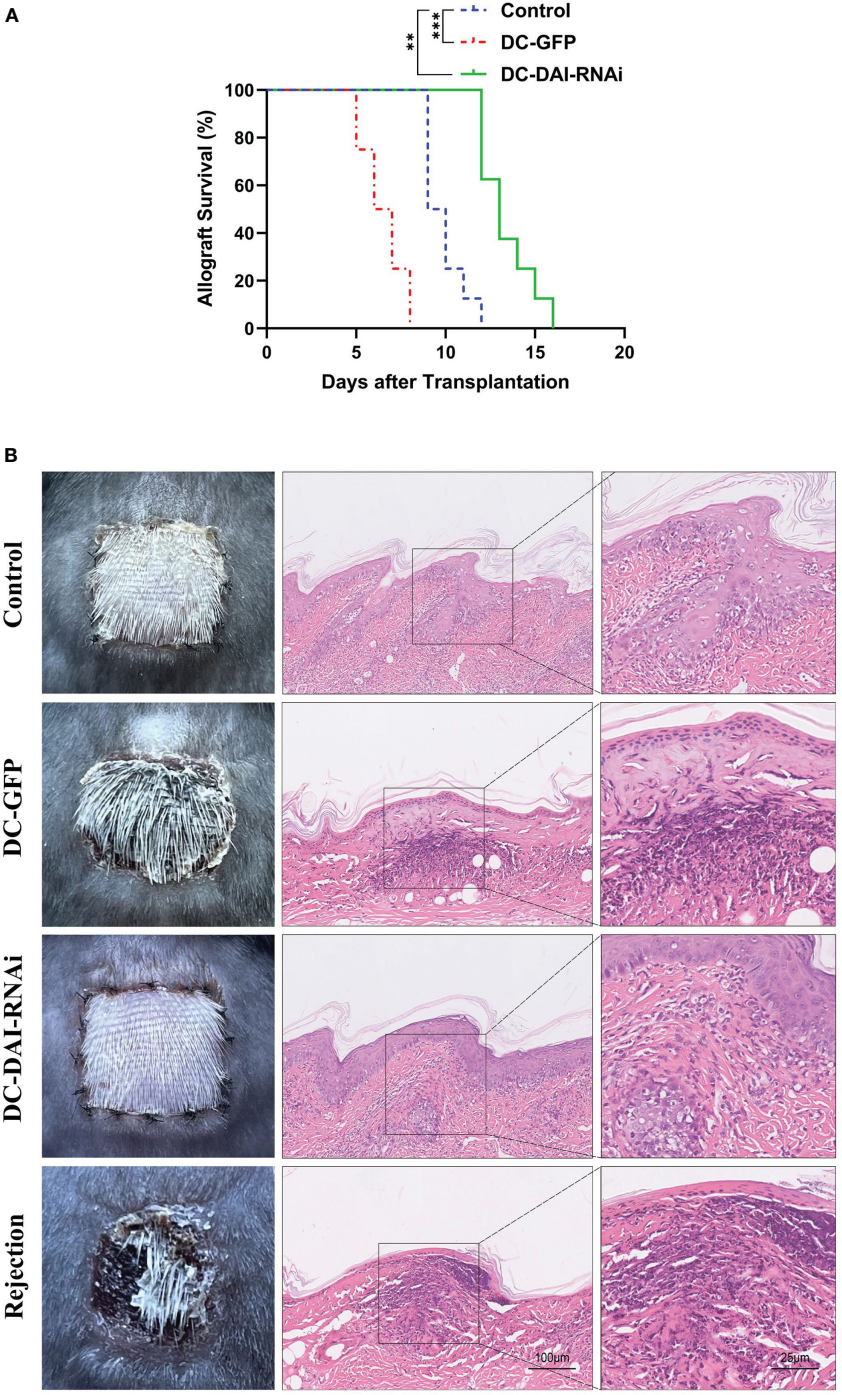
DCs transduced with AdV-DAI-RNAi-GFP prolonged the survival of the skin allograft. **(A)** Survival curves of skin allografts in the PBS control, DC-GFP, and DC-DAI-RNAi groups. Allograft survival among groups was compared with Kaplan–Meier analysis (**P < 0.01, ***P < 0.001, n=8). **(B)** On POD 7, skin allografts were removed from each group and stained with hematoxylin–eosin (HE). Skin allografts in the DC-DAI-RNAi group showed better survival with complete tissue structure and less lymphocyte infiltration (× 200, n=5).

Similarly, we observed a significantly prolonged survival time of skin allografts in the DC-DAI-RNAi group compared to other groups (**Figure 5A**). Morphologically, skin allografts in the DC-DAI-RNAi group had better survival than other groups on POD 7. Subsequently, the skin allografts in each group were removed for HE staining. The control and DC-GFP groups exhibited varying degrees of hair follicle destruction, dermal and subcutaneous tissue edema, and lymphocytic infiltration (**Figure 5B**). However, these were markedly alleviated in the DC-DAI-RNAi group.

### 3.4 DC-DAI-RNAi promotes Treg cell differentiation and induces immunosuppressive cytokines secretion *in vivo*

**Figure 6:**
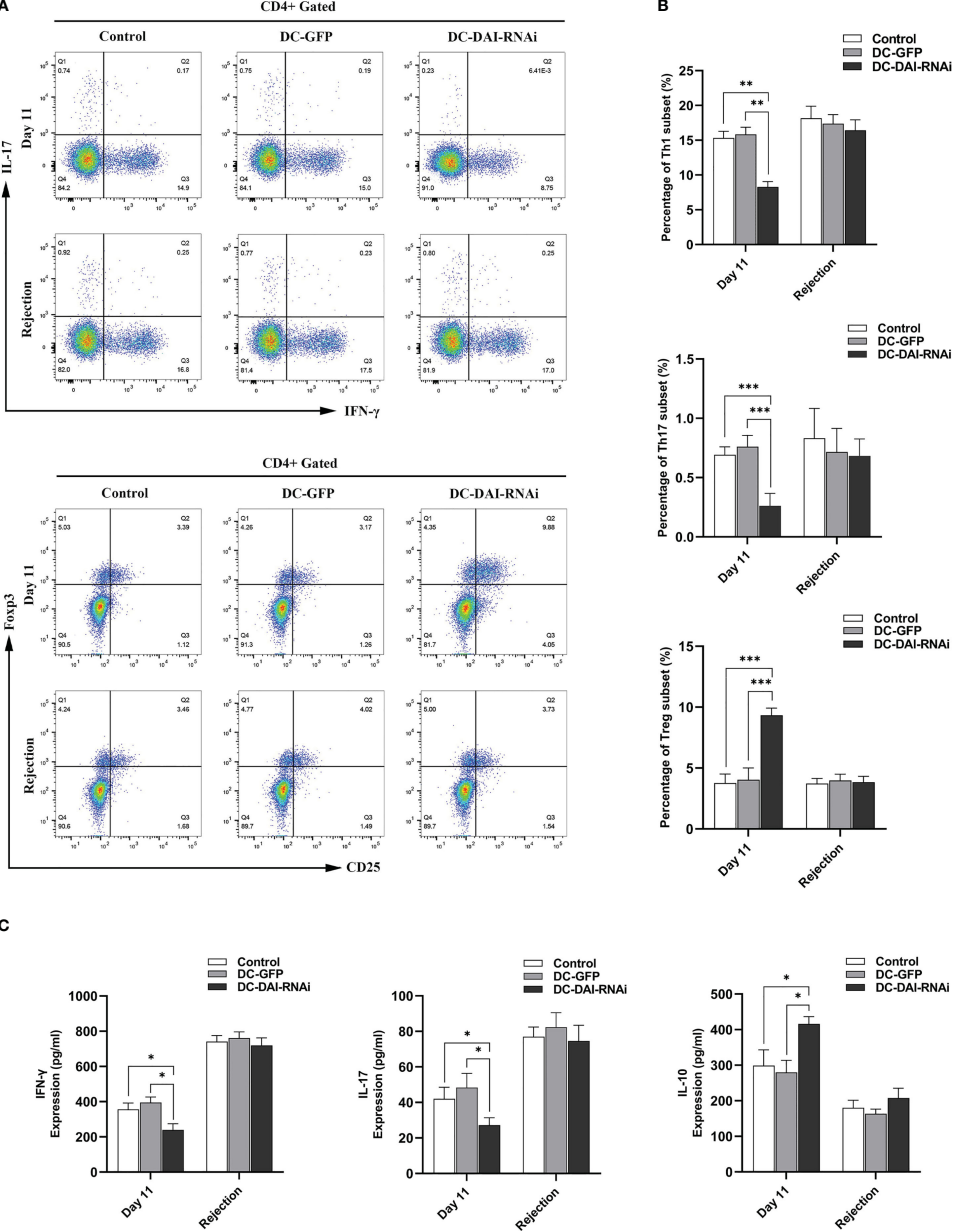
DC-DAI-RNAi reduced the populations of Th1 and Th17 cells and their secreted cytokines while induced Treg cell expansion and increased the IL-10 level in islet transplantation recipient mice. **(A, B)** The proportions of Th1, Th17, and Treg cells in the spleens of the PBS control, DC-GFP, and DC-DAI-RNAi groups on day 11 after islet transplantation and on the day of allograft rejection (**P < 0.01, ***P < 0.001, n=5). **(C)** Cytokine levels in serum were analyzed on day 11 after islet transplantation and on the day of allograft rejection. Data represent the mean ± SD (*P < 0.05, n=5).

We tested the proportion of T cell subsets and their secreted cytokines in the murine islet transplantation model to determine the immunoregulatory mechanisms by which DC-DAI-RNAi prolonged allograft survival. Splenic single cell suspension of recipient mice in each group were collected on POD 11 and on the day of allograft rejection, and then analyzed by flow cytometry after being stained with fluorescence-conjugated monoclonal antibodies. The results indicated that the proportions of Th1 and Th17 cells in the DC-DAI-RNAi group decreased significantly, whereas the proportion of Treg cells increased when compared with the other groups (**Figures 6A, B**). Meanwhile, blood samples from recipient mice retro-orbital sinus were collected on POD 11 and on the day of allograft rejection, and serum levels of immunostimulating cytokines IL-17 and IFN-γ and immunosuppressive cytokine IL-10 were measured by ELISA. The results showed that the IL-10 level in the DC-DAI-RNAi group was significantly higher than that in the DC-GFP group (P < 0.05), while IL-17 (P < 0.05) and IFN-γ (P < 0.05) levels were significantly lower in the DC-DAI-RNAi group compared to the other groups (**Figure 6C**).

## 4 Discussion

The present study demonstrated that DC-DAI-RNAi inhibited the expression of major co-stimulatory molecules and MHC-II, exhibited strong phagocytic ability, secreted high levels of immunosuppressive cytokines and low levels of immunostimulating cytokines, and negatively regulated T cell function. *In vivo*, DC-DAI-RNAi prolonged islet and skin allograft survival by inducing Treg cell expansion. We used a genetically modified adenovirus vector to improve the transduction efficiency of DCs (16), and the high expression of GFP by DCs indicated a high transduction efficiency, which made inhibition of DAI possible.

Allograft rejection is a complex process involving an array of events, central to which is the T-cell-mediated adaptive immune response initiated by activated DCs (17). PRRs are expressed mainly on APCs and initiate signaling pathways that trigger innate immunity, which induces DCs maturation and activation (18–20). DNA sensors belong to PRRs. DAI is the first reported cytosolic DNA sensor that contributes to the recognition of multiple types of nucleic acids and enhances the innate immune response by driving inflammatory signaling and a form of inflammatory cell death known as PANoptosis (21–24). It has been reported that DAI plays a crucial role in the maturation and activation of DCs during viral infections. Rahmatpanah et al. found that DAI is significantly up-regulated in DCs during defense against respiratory viruses by secreting high levels of type I interferons (IFN) (11). In a recent study conducted by Karki et al., the authors found that an increase in the severity of COVID-19 was associated with inflammatory cell death triggered by DAI up-regulated in activated immune cells, including DCs (12). To better understand the role of DAI in DCs maturation and activation, we explored the consequences of DAI inhibition in DCs. Our results revealed that inhibition of DAI could restrain LPS-induced DCs maturation, which was characterized by inhibition of major co-stimulatory molecules and MHC-II expression, increased phagocytic activity, and a decreased ability to stimulate T cell proliferation. DCs are recognized as the most potent stimulators of T cells, and the function of T cells is largely dependent on the cytokines secreted by DCs (25). Our results showed that the DAI-deficient DCs secreted higher levels of immunosuppressive cytokines and lower levels of immunostimulating cytokines. In addition, cytokine secretion levels in MLR supernatants further confirmed the immunosuppressive properties of DC-DAI-RNAi.

Previous studies have shown that inhibition of DCs maturation can promote the survival of allografts (26–30), and we validated the immunoprotective effect of DC-DAI-RNAi on islet and skin allografts in mice. In addition to allograft survival time, biological differences and physiological function of allografts were also compared among groups. Our results showed that DC-DAI-RNAi significantly prolonged the survival times of both islet and skin allografts, while DC-GFP exaggerated allograft rejection. Consistent with these findings, IPGTT performed in mice receiving islet transplantation, and pathological assessments of both islet and skin allografts also revealed a better survival status of allografts in the DC-DAI-RNAi-treated group. The aforementioned results demonstrated that inhibition of DAI expression could exert an immunoprotective effect on allografts in mice by restraining DCs maturation.

Subsequently, we further explored the immunoregulatory mechanisms of DC-DAI-RNAi *in vivo*. It is recognized that CD4^+^ T cell-mediated cellular immunity plays a vital role in allograft rejection, and their immunological properties differ by different subsets: Th1 and Th17 cells typically promote inflammatory immune responses, while Treg cells can inhibit immune responses (17). The directional differentiation of CD4^+^ T cells largely depends on the cytokine pattern and expression level of APCs co-stimulatory molecules. Studies have shown that imDCs can promote T cell differentiation into Treg cells while inhibiting Th1 and Th17 cell-driven rejection, thus promoting the survival of allografts (28, 30). Consistently, we observed an increase of Treg cells and a decrease of Th1 and Th17 cells in the DC-DAI-RNAi group of islet transplantation model, which were closely associated with alleviation of allograft rejection in our models. INF-γ and IL-17 are common immunostimulating cytokines that contribute to transplant rejection, while IL-10 is a well-known immunosuppressive cytokine that plays an important role in immune tolerance. Hence, we also investigated the regulatory effect of DC-DAI-RNAi on the secretion levels of cytokines in serum. As expected, we observed a significant increase in IL-10 level and a reduction in both INF-γ and IL-17 levels in the DC-DAI-RNAi group.

Notably, though injection of DC-DAI-RNAi prior to transplantation prolonged islet and skin allograft survival, allograft rejection eventually occurred. This may be related to decreased DC-DAI-RNAi over time and the continuous generation of protogenetic DCs in recipients. It remains to be explored whether persistent administration of DC-DAI-RNAi may induce persistent and stable immune tolerance. Besides, in our study, BMDCs were transduced with the recombinant adenovirus vector AdV-DAI-RNAi-GFP (DC-DAI-RNAi) or AdV-GFP (DC-GFP) at a relatively high MOI of 1:100 for 48 h to ensure high transduction efficiency, which may have affected the cell status of immature DC-GFP and DC-DAI-RNAi to a certain extent, and thus affected their therapeutic effects *in vivo*. This might be a technical limitation of our study. In future experiments, we plan to use specific DAI-knockout mice to avoid potential interference factors and obtain more rigorous and comprehensive experimental verification. Meanwhile, given the fact that transplant rejection involves complex regulatory networks of the immune system, the exact mechanism by which DAI modulates DCs maturation and improves allograft survival also needs to be further explored.

## 5 Conclusions

Inhibition of DAI in DCs inhibits the expression of major co-stimulatory molecules and MHC-II, induces expansion of Treg cells, promotes the secretion of immunosuppressive cytokines, and thus prolongs islet and skin allograft survival in mice. Although further research is needed to fully elucidate the molecular and cellular mechanisms by which DAI modulates DCs function and then regulates transplantation immunity, our findings point to a new therapeutic approach to promote long-term allograft survival.

## Data availability statement

The original contributions presented in the study are included in the article. Further inquiries can be directed to the corresponding authors.

## Ethics statement

The animal study was reviewed and approved by Institutional Animal Care and Use Committee (IACUC) of Sun Yat-sen University.

## Author contributions

PC and QJ contributed equally to this work and should be considered as co-first authors. PC analyzed the data, interpreted the results, and drafted the manuscript of the work; PC, QJ and ZF constructed the animal models; PC, QJ and RD analyzed the data; PC, QJ and YM interpreted the results; YM and RD designed the work and revised it critically. All authors contributed to the article and approved the submitted version.
